# An isolated ruptured spinal aneurysm presents with a thalamic Infarct: case report

**DOI:** 10.1186/s12883-021-02055-5

**Published:** 2021-02-03

**Authors:** Alexander Tenorio, Brandon B. Holmes, Adib A. Abla, Matthew Amans, Karl Meisel

**Affiliations:** 1 Department of Neurology, University of California, San Francisco, CA USA; 2 Department of Neurosurgery, University of California, San Francisco, CA USA; 3 Department of Neurointerventional Radiology, University of California, San Francisco, CA USA

**Keywords:** Posterior spinal aneurysm, Subarachnoid hemorrhage, Thalamic infarct

## Abstract

**Background:**

Isolated spinal artery aneurysms are extremely rare, and their pathogenesis, clinical presentation, and treatment strategies are poorly established. We report only the second case of a patient with an isolated posterior spinal aneurysm and concurrent left thalamic infarct and review the literature to help clarify treatment strategies of isolated spinal aneurysms.

**Case presentation:**

A 49-year-old patient presented with acute onset walking difficulty followed by diaphoresis, back and abdominal pain, and paraplegia. Imaging was notable for a hemorrhagic spinal lesion with compression at T12 through L4 and an acute left thalamic infarct. Surgical exploration revealed an isolated posterior spinal artery aneurysm. The aneurysm was surgically resected and the patient had partial recovery six months post-operatively.

**Conclusions:**

Isolated posterior spinal artery aneurysms of the thoracolumbar region are rare lesions that commonly present with abdominal pain, radiating back pain, and lower extremity weakness. Imaging may not provide a definitive diagnosis. The three primary treatment strategies are conservative management, endovascular treatment, or surgical resection. In patients with symptomatic cord compression, immediate surgical intervention is indicated to preserve neurologic function. In all other cases, the artery size, distal flow, morphology, and location may guide management.

## Background

Spinal artery aneurysms are a rare condition typically associated with vascular anomalies that generate local flow alterations such as fistulas, arteriovenous malformations (AVM), and coarctation of the aorta. When found in the absence of these vascular pathologies, they are referred to as isolated spinal artery aneurysms and usually occur in the anterior spinal artery. Isolated spinal artery aneurysms arising from the posterior axis of the spinal cord are exceptionally rare with optimal medical and surgical management being poorly defined. We report a case of a patient with a spinal subarachnoid hemorrhage caused by rupture of an isolated posterior spinal artery aneurysm that was surgically corrected. The patient also presented with a concurrent thalamic infarct which has only been described in the literature once before [1].

## Case presentation

### Clinical findings

A 49-year-old patient with a history of vestibular schwannoma and epilepsy developed acute onset walking difficulty. Two days later, the patient presented to an outside hospital with diaphoresis, abdominal pain, back pain radiating to the thighs, and bilateral lower extremity paralysis. A brain MRI revealed an acute left thalamic infarct and a spine MRI found compression at T12 through L4 concerning for subarachnoid or subdural hemorrhage. The patient was transferred to our institution for further work-up and management. On our admission, examination revealed dense lower extremity paraplegia with anesthesia and absent rectal tone concerning for conus medullaris syndrome. Additionally, the patient had right hemi-body sensory loss of light touch and pinprick in the face, upper torso, and arm.

### Imaging

**Fig. 1:**
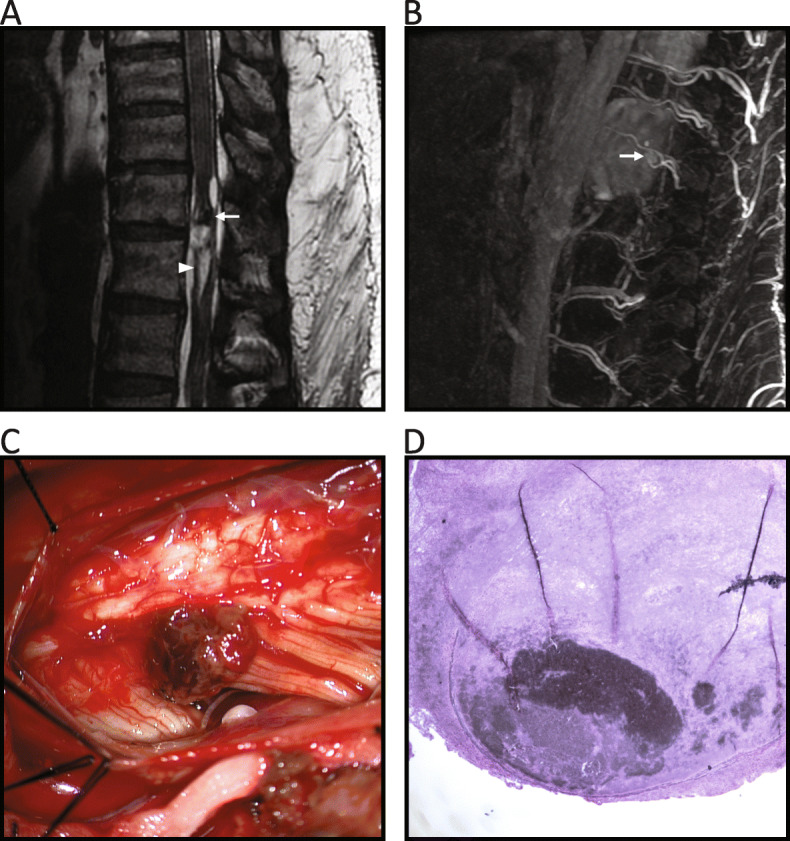
**a** Balanced steady-state gradient recalled echo sequences demonstrate a 1.7 cm intradural cystic and hemorrhagic mass within the spinal cord at thoracic levels 11 and 12 (arrow). There is associated intradural hemorrhage (arrowhead). **b** Spinal magnetic resonance angiogram shows a saccular aneurysm (arrow). **c** Surgical photograph reveals the posterior spinal aneurysm as an extrinsic mass lesion. **d** Histopathology of the resected aneurysm demonstrates positive reticulin staining

MRI with MRA of the complete spine demonstrated an intradural cystic and hemorrhagic mass along the left ventrolateral pial surface at the T11-12 level. This lesion was associated with extensive intradural hemorrhage with inferior extension into the lumbar spine (Fig. 1). The imaging findings were highly suggestive of subarachnoid and subdural hemorrhage initially thought to be due to a cavernous malformation vs. AVM. Brain MRI illustrated a T2 FLAIR hyperintense signal within the left thalamus consistent with an acute thalamic infarct. Conventional spinal angiography was negative for evidence of spinal aneurysm.

### Surgical Approach and Pathology

The following day, a laminectomy was performed at levels T10- L1. The dura was opened and blood was immediately visualized and evacuated from the subdural space. The dura was retracted laterally with subarachnoid blood becoming apparent. The arachnoid was opened and the subarachnoid blood was irrigated and removed using suction. At the left lateral aspect of T11 and T12, there was a hematoma compressing the spinal cord in the subdural space, which was also evacuated with suction. After removal of the hematoma, a nodular vascular lesion at the posterolateral spinal cord likely emanating from the posterolateral spinal artery was visualized, excised, and sent to pathology (Fig. 1c).

Surgical pathology demonstrated a thin segment of vessel wall, positive trichrome stain, and several peripheral reticulin fibers confirming the diagnosis of a spinal aneurysm (Fig. 1d). Post-operatively, the patient had improved sensation and gradually regained strength in the lower extremities. The patient was discharged to the transferring hospital five days after surgery. Six months post-operatively, the patient’s sensation and strength had improved but remained unable to walk with movement in the lower extremities limited to the toes.

### Left thalamic infarct

The left thalamic infarct was treated with standard post-stroke management. The patient was kept permissively hypertensive and Atorvastatin was initiated. HgbA1c was within normal range and Aspirin was started on post-operative day number 5; DAPT was not initiated given spinal SAH. The patient underwent a transthoracic echocardiogram and MRA of the neck, which were both unremarkable. The hemi-body sensory loss gradually improved and sensation was back to near baseline at discharge five days later.

## Discussion and conclusion

We present a case of a ruptured posterior spinal artery aneurysm with a concurrent left thalamic infarct. This combination of pathologies led to cauda equina syndrome with hemibody sensory loss. To our knowledge, this is the second reported case of an isolated spinal artery aneurysm associated with a left thalamic infarct.

The pathophysiology of isolated spinal artery aneurysms is still under investigation. The majority are associated with vascular malformations, with increased flow through the vessel leading to aneurysm formation [2]. In the absence of a malformation (i.e. isolated spinal aneurysms), dissection has been a proposed mechanism proven by histopathology [3]. These dissections often arise in the setting of a condition that weakens the vessels, such as connective tissue disorders and autoimmune disease [2]. In our case, surgical examination revealed a ruptured thrombosed aneurysm without evidence of dissection on histopathology.

The natural history of isolated spinal aneurysms is unclear due to its rarity. Through a literature review, we found 39 cases of isolated spinal aneurysms from 2011 to 2019 (Table 1). Kim et al. previously found 43 cases as of 2010 [3], bringing the total number to 83 when including our case. Our patient initially presented with radiating back pain, abdominal pain, and lower extremity weakness. This is consistent with previous reports, as the most common symptoms in thoracolumbar spinal aneurysms are sudden onset back pain, weakness, meningism, and abdominal pain [3]. A hemorrhagic lesion was also present on initial imaging, which was confirmed to be a ruptured aneurysm on surgical examination. Hemorrhage occurs at a very high rate in patients with spinal aneurysms associated with AVMs, with one review finding a 100 % incidence in 12 patients [4]. Although the data for isolated spinal aneurysms is limited, they are also likely to present with rupture and hemorrhage. In Kim *et. al.’s* review, 36 (84 %) presented with rupture [3].

**Table 1:** Demographic and Clinical Data of Isolated Spinal Aneurysms (2011–2019)

No.	Author/Year	Age/Sex	Co-morbidities	Location	Initial Presentation	Imaging Findings	Treatment	Outcome
1	Iihoshi 2011 [17]	60/F	-	T11	Headache, back pain, nausea	Spinal and Intracranial SAH	Conservative	Resolution
2	Kim 2012 [3]	52/M	Right acoustic neuroma, HTN, Meningoencephalitis	T7	Abdominal pain, headache, back pain radiating to LE	T7 Intradural extramedullary enhancing lesion	Embolization	Resolution
3	Shankar 2012 [18]	72/F	-	L2	Back pain radiating to LE	T12-L1 Lesion	Embolization	Improved
4	Takashima 2012 [19]	84/M	-	C1	Quadriplegia	Intramedullary C1 Hematoma	N/A	Death from respiratory dysfunction
5	Tanweer 2012 [20]	67/F	HTN, Atrial Fibrillation	T11	Back pain, Acute paraplegia and sensory loss	Spinal and Intracranial SAH	Embolization	Improved
6	Seerangan 2012 [21]	47/M	Intracranial aneurysms, ESRD, ADPKD	T7-T10	Lower extremity weakness, bowel/bladder disturbances	Intracranial and Spinal SAH	Resection	Minimal Recovery
7	Sato 2012 [22]	67/F	HTN, Dyslipidemia	T8 and T10	Acute back pain, paraparesis	T8 and T10 intradural masses, spinal infarction, spinal SAH	Conservative	Resolution
8	Van Es 2013 [23]	62/F	None	T12	Headache, back pain, walking difficulty	Spinal SAH	Resection	-
9	Van Es 2013 [23]	68/M	-	T4	Intrascapular back pain radiating to lumbar region, Headache, Nausea	T4 Hyperdense nodular lesion	Conservative (patient refusal)	Resolution
10	Marovich 2013 [24]	58/M	None		Cervico-thoracic back pain	C8-T6 Extradural Hemorrhagic Lesion	Resection	-
11	Yang 2013 [25]	47/M	-	ASA-cervical region	Neck Pain	Cranial SAH, IVH, ASA aneurysm	Conservative	Death from End-Stage Bile Duct Cancer
12	Son 2013 [26]	45/F	None	L1	Headache, back pain, nausea	Spinal and Intracranial SAH	Conservative	Resolution
13	Santana-Ramirez 2013 [27]	1/F	-	C3-C6	Quadriparesis, neck pain	C3-C6 intramedullary lesion	Resection	Improved
14	Pahl 2014 [28]	43/F	None	Cervi-medullary junction	Headache and vomiting	Intracranial SAH, IVH	Conservative	Resolution
15	Romero 2014 [29]	37/F	-	T4	Thoracic/cervical pain, headache	Spinal and Intracranial SAH	Conservative	Resolution
16	Romero 2014 [29]	72/F	HTN, DM, CRF	T10	Cervical pain, headache, neck stiffness	Spinal and Intracranial SAH	Conservative	Improved
17	Bell 2014 [30]	68/F	-	T5	Severe back pain	Thoracic intradural lesion and lumbar SAH	Resection	-
18	Johnson 2015 [31]	Teenager/-	None	C5-C6	Headache, neck pain, nausea	C5-C6 enhancing nodular lesion	Resection	Resolution
19	Ronchetti 2015 [32]	51/F	-	T1-T4	Neck pain, headache, bilateral leg numbness, difficulty walking	Thoracic extramedullary hemorrhage	Resection	Resolution
20	Ronchetti 2015 [32]	68/M	-	T1	Mid-back pain radiating to neck	Intracranial SAH, Cervico-Thoracic SAH	Embolization	Resolution
21	Sung 2015 [33]	74/M	HTN, Ischemic Heart Disease	T1	Chest pain radiating to neck/back	Intracranial and Spinal SAH	Resection	Resolution
22	Horio 2015 [1]	84/M	Right Thalamic Infarct	T12	Left Hemiplegia	Spinal SAH	Resection	Improved
23	Takata 2016 [34]	72/F	None	T9	Acute back pain	T4-T10 SAH	Resection	Resolution
24	Doberstein 2016 [35]	59/M	Parkinson’s, T-cell lymphoma	T11	Back spasms, walking difficulty	T6-L2 Hyperintensity	Conservative	Resolution
25	Ikeda 2016 [36]	54/M	-	T10	Severe back pain, vomiting	Spinal SDH and SAH	Resection	Resolution
26	Hill 2016 [37]	53/M	HBV, HCV	T9	Paraplegia	C7-T1 intradural lesion	Resection	Death from medical complications
27	Roka 2016 [38]	30/F	None	Cervical ASA	Headache, vomiting, vertigo	IVH	Conservative	Resolution
28	Kogan 2017 [39]	58/F	-	T2	Headache radiating to neck and upper back, nausea, vomiting	T1-T5 hyperintensity	Resection	Resolution
29	Dabus 2018 [9]	60 s/-	-	Cervico-medullary Junction	Headache and neck pain	SAH	Conservative	Resolution
30	Dabus 2018 [9]	30 s/-	-	Cervical	Back Pain	SAH	Conservative	Resolution
31	Dabus 2018 [9]	60 s/-	-	Mid-Thoracic	Back pain and LE paresthesia	Intramedullary Hemorrhage	Conservative	Resolution
32	Dabus 2018 [9]	50 s/-	-	Lower Thoracic	Back pain and LE paresthesia	SDH	Conservative	Resolution
33	Aljuboori 2018 [40]	78/M	HTN, PVD, HLD, CAD	T9	Acute Back Pain, LE weakness	T9 aneurysm, cord compression	Resection	Resolution
34	Aguilar-Salinas 2018 [41]	54/F	HTN	T10	Headache, back pain, nausea, vomiting	Spinal SAH with cord compression	Conservative	Improved
35	Ren 2018 [42]	57/F	N/A	C1	Severe headache	Intracranial SAH	Resection	Resolution
36	Ren 2018 [42]	27/F	N/A	L1	Bilateral LE pain/numbness	Lesion at Conus Medullaris	Resection	Resolution
37	Morozumi 2018 [43]	9/M	None	C7-T1	Back pain, paralysis	C7-T1 Lesion with hemorrhage	Resection	Resolution
38	Simon-Gabriel 2018 [44]	65/M	HTN, Hypercholesterolemia, Tachyarrhythmia	Cranio-cervical ASA	Neck stiffness	SAH with tamponade of 4th ventricle	Flow diverting stent	Resolution
39	Priola 2019 [45]	54/F	None	T3	Upper thoracic back pain radiating to neck and head	Cervico-thoraco-lumbar spine Hematomas	Resection	Resolution

*HTN Hypertension, *DM* Diabetes Mellitus, *CRF* Chronic Renal Failure, *ESRD* End Stage Renal Disease, *ADPKD* Autosomal Dominant Polycystic Kidney Disease, *PVD* Peripheral Vascular Disease, *HLD* Hyperlipidemia, *CAD* Coronary Artery Disease, *HBV* Hepatitis B Virus, *HCV* Hepatitis C Virus, *LE* Lower Extremities, *SAH* Subarachnoid Hemorrhage, *IVH* Intraventricular Hemorrhage, *ASA* Anterior Spinal Artery

Search Strategy:

• Google scholar: isolated AND spinal AND aneurysm

• Date range: 2011–2019

• 990 results reviewed

The treatment strategy for spinal aneurysms is also controversial, with three primary strategies being used: (1) surgical resection, (2) endovascular treatment, or (3) conservative management [3]. Some authors have advocated for conservative management given their experience of spontaneous regression [5, 6]. They suggest that compression of the spinal cord from the aneurysm or surrounding blood should be the only indications for intervention. Karakama et al. presented a case of a ruptured anterior spinal artery aneurysm treated conservatively and recommended strict blood pressure control and follow-up imaging to monitor for progression [7]. This aneurysm was located in the anterior cervical region and conservative management was selected due to concern of disturbing blood flow of the parent artery. Longatti et al. presented a case of a patient with multiple anterior spinal artery aneurysms that were treated conservatively [8]. This strategy was selected due to the absence of cord compression, small artery size, and presence of distal flow. Dabus et al. presented a case series of four patients with dissecting spinal aneurysms that were treated conservatively, with the size of the parent artery and tissue supplied being the main determinants [9]. In these cases, the artery size, presence of distal flow, fusiform morphology, and surgical access were factors that favored a conservative approach.

Despite the successful cases with a wait-and-see approach, the overall occurrence of this condition is too limited to draw definite conclusions. There have been five reported cases of a patient dying without surgical intervention, with re-bleeding being the most common cause [10–14]. Early surgical intervention has been proposed for posterior spinal aneurysms due to its superficial location and ability to be resected safely, with all cases resulting in complete resolution [15]. Anterior spinal aneurysms are more difficult to access surgically and provide major blood supply to the spinal cord, which can lead to severe neurologic sequalae if interrupted. In our case, the patient presented with progressive symptoms of spinal cord compression, most notably paraplegia. Imaging did not provide a definitive diagnosis, so prompt surgical intervention was indicated to decompress the spinal cord and preserve neurologic function.

Whether the spinal aneurysm was the source of the left thalamic infarct or an incidental finding is difficult to determine. To the best of our knowledge, no cases in the literature have reported a spinal aneurysm as a cause of a cerebral infarct, although one case exists linking a subarachnoid hemorrhage with a spinal cord infarct [16]. This suggests that blood products in the subarachnoid space might lead to secondary ischemia at a distance, and indeed, multiple mechanisms have been postulated that connect subarachnoid blood with micro and macro circulatory failure. For example, release of intracellular material such as oxyhemoglobin from red blood cell lysis can lead to altered vessel dynamics through inactivation of nitric oxide, over-expression of endothelin peptides, and under-expression of prostacyclin leading to platelet aggregation [17]. Should these mechanisms have contributed to the thalamic infarct observed in our patient, it is unclear why the arteries supplying the thalamus would have been particularly susceptible. Along these lines, MRA of the head and neck did not reveal evidence of vasculopathy. An additional mechanism to link these two pathologies is hypertension. Hypertension is common following subarachnoid hemorrhage likely due to pain, anxiety, and sympathetic activation. Thalamic infarcts are most often caused by microvascular disease with hypertension accounting for ~ 68 % of cases [18]. It is noteworthy that in the 83 cases of isolated spinal artery aneurysms reported in literature, two cases with concurrent thalamic infarcts have now been observed which is statistically unexpected. Further investigations are needed to determine whether there is a mechanistic link between these two pathologies, and future cases may benefit by pursuing cerebral angiography at the same time of spinal angiography.

In conclusion, isolated spinal artery aneurysms are an exceedingly rare occurrence, particularly in the posterior axis of the spine. Imaging with MRI/CT and angiogram may not provide a definitive diagnosis. We agree with previous authors that in a patient with a posterior spinal aneurysm and symptoms of spinal cord compression, prompt surgical intervention is warranted. Finally, further studies are needed to understand the possible interaction between subarachnoid blood products and cerebral infarction.

## Data Availability

All data generated or analyzed during this study are included in this published article and its supplementary information files.
